# Identification of Apo-A1 as a biomarker for early diagnosis of bladder transitional cell carcinoma

**DOI:** 10.1186/1477-5956-9-21

**Published:** 2011-04-17

**Authors:** Hongjie Li, Changying Li, Huili Wu, Ting Zhang, Jin Wang, Shixin Wang, Jiwu Chang

**Affiliations:** 1 Department of Pathophysiology, HeBei United University, Tangshan, China; 2 Research Laboratory for Tumor Immunity of Tianjin Urological Institute, Second Hospital of Tianjin Medical University, Tianjin, China; 3 Tianjin Key Laboratory of Biomarkers for Occupational and Environmental Hazard of Chinese People's Armed Police Forces Medical College, Tianjin, China

## Abstract

**Background:**

Bladder transitional cell carcinoma (BTCC) is the fourth most frequent neoplasia in men, clinically characterized by high recurrent rates and poor prognosis. Availability of urinary tumor biomarkers represents a convenient alternative for early detection and disease surveillance because of its direct contact with the tumor and sample accessibility.

**Results:**

We tested urine samples from healthy volunteers and patients with low malignant or aggressive BTCC to identify potential biomarkers for early detection of BTCC by two-dimensional electrophoresis (2-DE) coupled with mass spectrometry (MS) and bioinformatics analysis. We observed increased expression of five proteins, including fibrinogen (Fb), lactate dehydrogenase B (LDHB), apolipoprotein-A1 (Apo-A1), clusterin (CLU) and haptoglobin (Hp), which were increased in urine samples of patients with low malignant or aggressive bladder cancer. Further analysis of urine samples of aggressive BTCC showed significant increase in Apo-A1 expression compared to low malignant BTCC. Apo-A1 level was measured quantitatively using enzyme-linked immunosorbent assay (ELISA) and was suggested to provide diagnostic utility to distinguish patients with bladder cancer from controls at 18.22 ng/ml, and distinguish patients with low malignant BTCC from patients with aggressive BTCC in two-tie grading system at 29.86 ng/ml respectively. Further validation assay showed that Apo-A1 could be used as a biomarker to diagnosis BTCC with a sensitivity and specificity of 91.6% and 85.7% respectively, and classify BTCC in two-tie grading system with a sensitivity and specificity of 83.7% and 89.7% respectively.

**Conclusion:**

Taken together, our findings suggest Apo-A1 could be a potential biomarker related with early diagnosis and classification in two-tie grading system for bladder cancer.

## Background

Bladder cancer is one of the tumors associated with the highest morbidity and mortality. It is the second most common urological cancer, clinically characterized by high recurrent rates and poor prognosis once tumors invade the lamina propia [1]. Cystoscopy and cytology are currently considered the 'gold standards' for the identification and monitoring for recurrence or progression of bladder cancer. Frequent cystoscopies facilitate the treatment of recurrences at an early stage, thereby potentially slowing the progression of the disease to muscle invasive disease. However, cystoscopy is an invasive, time-consuming and expensive examination and is not well-accepted for patients [2]. Urine cytology is a highly specific, noninvasive adjunct to cystoscopy that is quite sensitive in detecting high grade bladder cancers. However, it has poor sensitivity in detecting low grade disease, and its accuracy is dependent on the pathologists' experience [3]. Therefore, scientists are interested in identifying reliable non-invasive biomarkers that could be utilized in screening, leading to early detection and/or in predicting the progression of superficial tumors to invasive higher-stage lesions with high specificity and sensitivity.

Proteomic patterns in body fluids present new opportunities for the development of novel, highly sensitive diagnostic tools for early detection of cancer [4]. A major goal in the field of clinical proteomics is to identify disease biomarkers in biological fluids that can be measured relatively inexpensively for early diagnosis of disease. Most of the focus thus far has been on proteomics of blood serum or plasma [5]. Since urine is directly exposed by bladder epithelium, it is the important source of information for bladder cancers. Also, urine can be collected non-invasively in large amounts, which provides an attractive alternative to blood plasma as a potential source of disease biomarkers for bladder cancer. Two-dimensional electrophoresis (2-DE) has been the mainstay of electrophoresis technology for a decade and is the most widely used tool for separating protein mixtures such as in cell and tissue extracts or body fluids [6]. Mass spectrometry (MS) allows the analysis and identification of very small amounts of protein isolated from the gel. In the past 10 years, 2-DE followed by MS has been the primary technique for biomarker discovery in conventional proteomic analyses [7,8]. Several proteins in urine are measured as markers for bladder cancers as well as those in blood, such as bladder tumor antigen [9], nuclear matrix proteins [10] and fibrinogen degradation products [11].

A cornerstone in the investigation of bladder cancer is the recognition of the two phenotypic tumors: low malignant and aggressive BTCC [12,13], which suggested two-tie grading system in BTCC [14,15]. The low malignant BTCC, accounting for 70%-80% of the urothelial carcinomas, presents as superficial, papillary lesions which has a propensity to recur, but only infrequently progresses to muscle-invasive stage or metastasize. The pathological characteristic is low-grade/well-differentiated neoplasms, previously classified as grade I-II. If treated promptly, the 5-year survival rate of this variant can approach 90%. The aggressive BTCC, accounting for 20%-30%, presents as an invasive tumor at diagnosis, and has a very high risk of progressing to incurable distant metastases. Their pathological morphology is the high-grade lesion that begins as dysplasia or carcinoma *in situ*, and occasionally as high-grade papillary carcinoma. Thus, BTCC appears to develop and progress via two distinctive phenotypic pathways with drastically different biological behavior and clinical outcome, which strongly supported by genetic analyses and clinical experimental evidence [16,17]. Early diagnosis and exact classification are two major challenges facing bladder cancer investigations. Currently, the molecular basis for these characteristics of these two phenotypic tumors is still unknown and no effective biomarkers found. Thus, we tend to identify new markers for classification in two-tie grading system in order to predicting both progression and treatment of BTCC.

The aim of this study was to identify the proteins whose expression is elevated in urine samples of patients with BTCC in comparison to that of healthy individuals and to find out the potential relationship between differentially expressed urinary proteins and the clinical diagnostic and classification of BTCC. Using the 2-DE approach with subsequent MS, we identified five differentially expressed proteins in urine samples from patients with BTCC in the comparison to healthy individuals. Moreover, our data strongly suggested Apo-A1 could be a potential biomarker for BTCC early diagnosis and clinical classification.

## Results

### Proteomics of urine samples from the healthy individuals and bladder cancer patients

**Figure 1:**
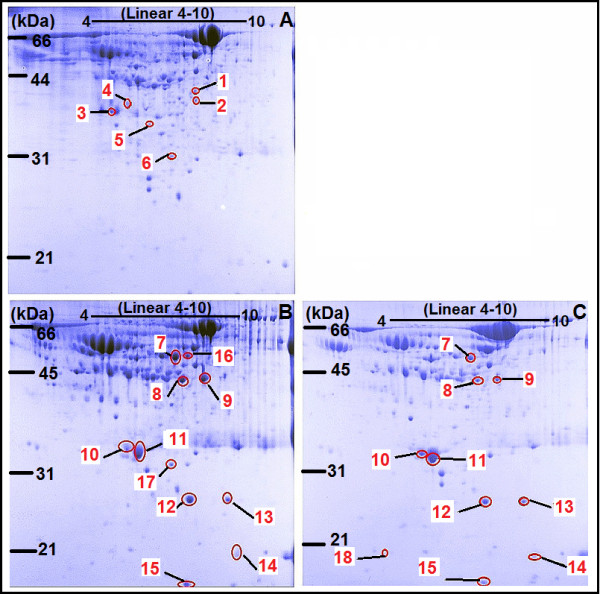
**2-DE profiles of urine samples**. Pooled urine samples from 24 healthy individuals (A), 24 patients with low malignant BTCC (B) and 16 patients with aggressive BTCC (C) were resolved on 2-DE gels. Protein spots were visualized using CBB. Spots with more than 10-fold difference of two matched spots from two different groups were numbered. The red arrows labeled with numbers indicate the position of the differentially expressed proteins.

To determine the changes in the protein expression pattern due to bladder cancer, 2-DE was performed on urine samples from patients with low malignant or aggressive BTCC, or healthy individuals as control. Protein spots were visualized using Coomassie Brilliant Blue (CBB) G-250. 2-DE images obtained from urine samples of healthy volunteers and patients with low malignant BTCC and aggressive BTCC respectively showed 613, 640 and 624 spots representing differentially expressed proteins (Figure 1). The majority of the spots located between molecular weight 10 kDa to 97 kDa in the pI range 4.5-7.0. 2-DE images obtained from the urine samples of sixteen normal volunteers revealed almost the same expression pattern of proteins. Several spots, which were present in urine samples of patients with bladder cancer, were not present in urine samples of healthy individuals.

If the relative protein content of two matched spots from every two groups (healthy individuals, patients with low malignant or aggressive BTCC) is different more than 10-fold, we considered it as a differentially expressed protein spot. According to this principle, 18 spots were identified, of which spots 1-6 were detected in the urine samples of healthy individuals; spots 7 and 8 were only present in the samples of BTCC patients; spots 16 and 17 were detected in the urine samples of patients with low malignant BTCC; spot 18 was only present in the urine samples of patients with aggressive BTCC.

### Identification of differentially expressed proteins by mass spectrometry

To further identify the proteins which differentially expressed among neoplastic versus non-neoplastic urine samples, relative protein spots were excised from CBB stained gels and identified by MS. Identified proteins are shown in Table 1, including protein name, accession number in NCBI, molecular weight (MW), isoelectric point (pI), MASCOT score, total ion score and location. Identification numbers in Table 1 matched with those associated to the 2-DE spots shown in Figure 1. In this study, 18 spots were found to be differentially expressed between urine samples of patients with bladder cancer and controls in a statistically significant manner. MS revealed 8 differential spots (spots 7-13 and 16), which represent five proteins (More than one spot revealed the presence of the same protein). These five proteins include fibrinogen (Fb), lactate dehydrogenase B (LDHB), apolipoprotein-A1 (Apo-A1), clusterin (CLU) and haptoglobin (Hp). Spots 7, 8 and 16 were identified to be different chains of fibrinogen, both spots 10 and 11 represented Apo-A1.

**Table 1:** Differentially expressed proteins identified by MS after 2-DE

Spot No.	Protein Name	Accession Number (NCBI)	MW(Da)	pI	MASCOT score	Total Ion Score C.I. %	Location
S7	fibrinogen gamma chain	gi|119625323	37714	5.87	177	100	T1,T2
S8	fibrinogen beta chain	gi|119625344	39711	6.95	137	100	T1,T2
S9	lactate dehydrogenase B	gi|119616854	36615	5.71	104	99.9988	T1,T2
S10	Apo-A1	gi|490098	28061	5.27	235	100	T1,T2
S11	Apo-A1	gi|490098	28061	5.27	207	100	T1,T2
S12	Clusterin	gi|42740907	52461	5.89	102	99.9565	T1,T2
S13	hp2-alpha	gi|296653	41499	6.25	115	98.9869	T1,T2
S16	fibrinogen gamma chain	gi|119625325	35480	6.07	100	100	T1

T1: the protein expression increased in urine samples from patients with low malignant BTCC;

T2: the protein expression increased in urine samples from patients with aggressive BTCC.

### Confirmation of protein expression by Western blot

**Figure 2:**
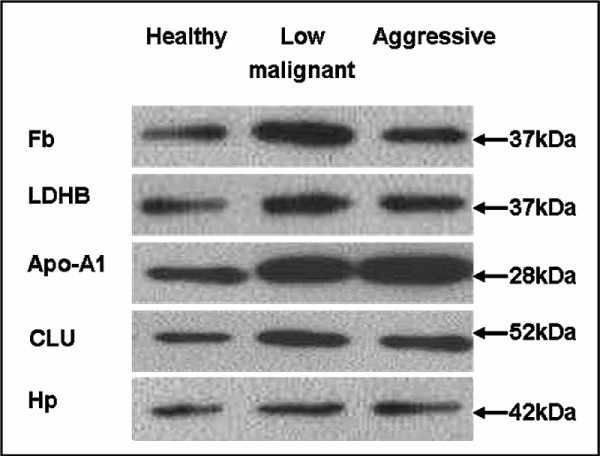
**Western blot validation of the proteins identified by 2-DE coupled with MS**. Fb, LDHB, Apo-A1, CLU and Hp expression in pooled urine samples from healthy individuals, patients with low malignant or aggressive BTCC were further detected by Western blot. Equal protein amount (25 μg) from each group was loaded onto 10% SDS-PAGE gels. Antibodies were accepted as detecting a single predominant band at the expected molecular weights.

To quantify the expression of these identified proteins, pooled urine samples were further examined by Western blot. The result showed that each protein displayed a single predominant band at the expected molecular weights (Figure 2). Fb, LDHB, Apo-A1, CLU and Hp were shown to be of 37 kDa, 37 kDa, 28 kDa, 52 kDa, 42 kDa, respectively. Fb, LDHB, Apo-A1 and CLU showed increasing trend in urine samples of patients with bladder cancer, compared with healthy control, while Hp expression didn't change significantly. Impressively, Apo-A1 expression level enhanced significantly in urine samples of patients with bladder cancer, particularly in aggressive BTCC group. Compared to healthy volunteers Apo-A1 expression was increased 5-fold and 11-fold in the urine samples of low malignant BTCC and aggressive BTCC respectively. Compared to low malignant BTCC group, the expression of Apo-A1 was increased by 3-fold in 85% of urine samples of aggressive BTCC.

### Apo-A1 expression in a series of independent urine samples

**Figure 3:**
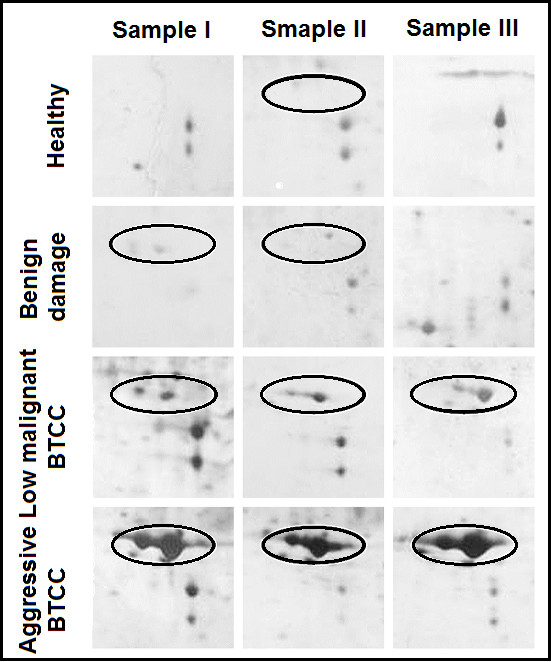
**2-DE profiles of Apo-A1 expression on independent series of urine samples**. Independent urine samples from healthy individuals, patients with bladder benign damages, patients with low malignant or aggressive BTCC were resolved on 2-DE gels and stained by sliver. Differentially expressed Apo-A1 was highlighted with an open oval which was further identified by MALDI-TOF/TOF-MS. Three representative gels (Sample I, II and III) of each group were shown.

Human Apolipoprotein A1 (Apo-A1) is the major protein component of high-density lipoprotein (HDL) in plasma [18,19]. Previous studies have reported the diagnostic value of Apo-A1 in early tumor detection in that increased levels of Apo-A1 were found in a variety of malignant tumors [20,21]. Giusti et al [20] reported that Apo-A1 precursor was significant up-regulated suggesting it could be a potential protein biomarker in the diagnostic classification of thyroid cancer. In the present study, the expression of Apo-A1 was increased in pooled urine samples of malignant patients compared to healthy individuals in Western blot. Therefore, Apo-A1 was further tested by 2-DE using a series of independent urine samples from healthy individuals, patients with bladder benign damages and those with low malignant or aggressive bladder cancers. Given many biomarkers could be less specific in the presence of benign genitourinary conditions [22,23]; we added urine samples of patients with bladder benign damages as control. Apo-A1 was detected in all urines samples (n = 40) of bladder cancers classified as low malignant and aggressive BTCC in two-tie grading system. But the expression in aggressive BTCC was apparently higher compared to low malignant BTCC. Apo-A1 was also found in some of urine samples of the patients with bladder benign damages and healthy volunteers; however, the expression was significantly low compared to BTCC group and there was no significant difference detected between these two groups (Figure 3). The result was consistent with findings by Western blot.

### Evaluation of Apo-A1 as a biomarker for BTCC diagnosis and classification

**Figure 4:**
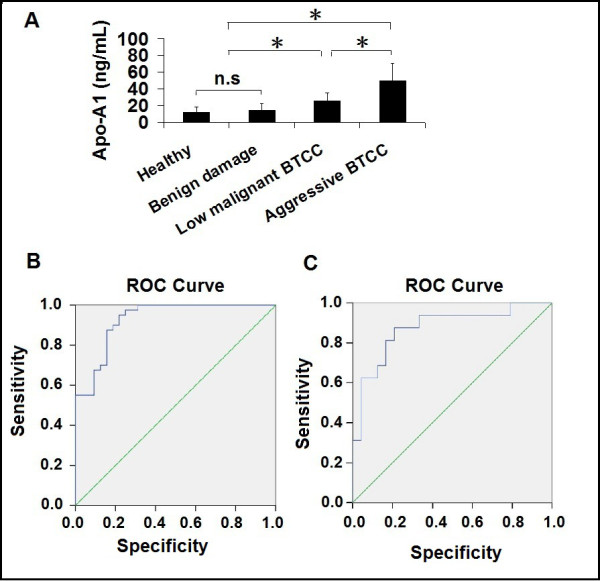
**Apo-A1 as a biomarker for predicting and classifying BTCC**. (A) Quantitative proteomic analysis of Apo-A1 in urine samples by ELISA. *P < 0.01, Student's *t*-test. (B) Urianry Apo-A1 distinguishes bladder cancer on independent series of urine samples of patients with BTCC and controls. ROC curve of urinary Apo-A1 as a detection marker for bladder cancer was based on a series of 72 urine samples. Among these, 40 had cancer-positive. The optimal cutoff was 18.22 ng/ml, and the AUC obtained was 0.928 (95% CI 0.870-0.986). (C) Urinary Apo-A1 classifies with a different series of urine samples of patients with low malignant or aggressive BTCC. ROC curve of urinary Apo-A1 as a detective classification marker for BTCC was based on a series of 40 urinary specimens, which consists of 24 low malignant BTCC and 16 aggressive BTCC. The optimal cutoff was 29.86 ng/ml, and the AUC obtained was 0.875 (95% CI 0.758-0.992).

Apo-A1 was further tested whether quantitative measurement could be utilized as a diagnostic tool to distinguish patients with bladder cancer from the healthy volunteers by ELISA. Once calibration curves were proven to be analytically optimal, Apo-A1 was measured in 72 urine samples by an ELISA according to the manufacturer's protocol. The result showed significant increased Apo-A1 expression in BTCC groups (*P <*0.01, Figure 4A). Considering sample categorization given by urinary cystoscopy, ROC analyses rendered a diagnostic accuracy of 0.928, measured by the area under the curve (AUC, *P *< 0.01, 95% confidence interval (CI) 0.870-0.986), at an 18.22 ng/ml cutoff (Figure 4B). Apo-A1 was also tested whether quantitative measurement could be utilized as a diagnostic tool to differentiate patients with low malignant BTCC and aggressive BTCC. Using the cystoscopy as the gold standard for urinary sample categorization, Receiver operating curve (ROC) analyses rendered a classified accuracy of 0.875, measured by the AUC (*P *< 0.01, 95% CI 0.758-0.992), at a 29.86 ng/ml cutoff (Figure 4C).

### Validation of Apo-A1 as a biomarker for BTCC diagnosis and classification

To further validate Apo-A1 as a potential biomarker for diagnosis and classification of bladder cancer, 156 urine samples from healthy individuals or patients with urinary system diseases were harvested randomly. Apo-A1 levels were measured by ELISA and a clinical diagnosis and classification were made according to the optimal cutoff values suggested by ROC curves in Figure 4B and 4C. Using the histopathological result as the gold standard, Apo-A1 can diagnosis BTCC with a sensitivity and specificity of 91.6% and 85.7% at 18.22 ng/ml cutoff (Table 2A); also it can classify BTCC in two-tie grading system with a sensitivity and specificity of 83.7% and 89.7% at 29.86 ng/ml cutoff (Table 2B).

**Table 2:** Validation of Apo-A1 as biomarker for diagnosis (A) and classification (B) of BTCC

A		
**Gold standard**	**Apo-A1 expression**	
	
	**>18.22 ng/ml**	**<18.22 ng/ml**

BTCC (107)	98	9
Benign damage (49)	7	42

B		

Gold standard	Apo-A1 expression	
	
	>29.86 ng/ml	<29.86 ng/ml

Aggressive BTCC (49)	41	8
Low Malignant BTCC (58)	6	52

## Discussion

Bladder cancer is the second most common urological tumors, clinically characterized by high recurrent rates and poor prognosis [1]. Thus, there is a clear need to identify new tumor specific biomarkers for early diagnosis and accurate classification of BTCC to make an appropriate therapeutic plan for cancer patients. Urine diagnostics for bladder cancer is an important diagnosis for years. Urine is directly exposed by bladder epithelium and is the important source of information for bladder cancers and readily collected. Here, we have performed urine proteomic analysis using 2-DE coupled with mass spectrometry. This represents a promising approach for the identification of tumor biomarkers aiming at the detection of the presence of bladder cancer and definite classification. Several proteins were found to be differentially expressed in a significant manner in urine samples of patients with bladder cancer as compared to controls, or between different classes or stages, as confirmed by gold standard diagnostic methods. In this study, we selected 18 spots with 10-fold variation between 3 groups for MS identification. Some spots were not included into the analysis because the MASCOT scores of those spots were below the cutoff score preset in the database or were identified as unnamed protein product. Totally, we identified 8 spots which are belong to 5 proteins, including fibrinogen (Fb), lactate dehydrogenase B (LDHB), apolipoprotein-A1 (Apo-A1), clusterin (CLU) and haptoglobin (Hp). These proteins were differentially expressed in a significant manner in urine samples of patients with bladder cancer as compared to controls. We also identified specific biomarkers for classifying bladder cancer in two-tie grading system and found urinary measurement of Apo-A1 expression on independent series of urine samples served to distinguish not only patients with bladder cancer from controls, but also patients with low malignant BTCC from aggressive BTCC with high diagnostic accuracy.

One of the markers identified in this study, Apo-A1, is the major protein component of high-density lipoprotein (HDL) in plasma [19]. It is synthesized in the liver and intestine as a prepro-protein. Apolipoprotein levels reflect the difference in HDL protein concentration observed among healthy controls but are also influenced by other possible confounders such as age, alcohol intake, hormone use, sex, race, body mass index, and coronary artery disease. Apo-Al is well-known as a negative marker of inflammation; its concentration decreases more than 25% during inflammation. Apo-A1 has been shown to be a specific inhibitor of cytokine production by monocytes and macrophages in response to interaction with stimulated T cells. Apo-A1 is a constitutive anti-inflammatory factor, and the decrease in HDL-associated Apo-A1 level may be a signal of chronic inflammation progression. It was shown that Apo-A1 concentration in blood is reduced in different types of cancer [24,25]. Apo-A1 has been identified as a potential biomarker of ovarian cancer, colorectal cancer, and pancreatic cancer by using MS. However, controversial observations were also reported including up-regulation of Apo-A1 in a variety of malignant tumors of ovarian, liver, breast [20,21].

In this study, spots 10 and 11 were identified as Apo-A1 in the 2-DE gels followed by MS. In pooled urine samples analyzed, we found a significant gradient difference in the chemiluminescence signal detected by Western blot for Apo-A1 between groups of healthy individuals and patients with low malignant and aggressive BTCC. So the question of the possible use of Apo-A1 for diagnosis and classification of BTCC needs to be further addressed. Considering the expression levels in pooled samples do not reflect the expression level in individual sample, Apo-A1 was further analyzed by 2-DE using a series of independent urine samples. Given many biomarkers could be less specific in the presence of benign genitourinary conditions [22,23], we added urine samples of patients with bladder benign damages as control. According to the protein density visualized using silver, Apo-A1 showed very low expression in urine samples of healthy individuals and patients with bladder benign damages, while significant increase in urine samples of patients with bladder cancer, particularly in those diagnosed as aggressive BTCC. These results further suggest that Apo-A1 could be a potential biomarker for diagnosis and classification of BTCC.

So next, Apo-A1 was further tested whether quantitative measurement could be utilized as a diagnostic tool to distinguish patients with bladder cancer and controls and classify bladder cancer. ELISA was performed and the result showed there was no difference between healthy individuals and patients with bladder benign damages, while the difference between these two groups and BTCC group was significant (*P *< 0.01), suggesting Apo-A1 could be a potential biomarker to distinguish BTCC patients from healthy individuals and patients with bladder benign damages. Also, the difference between low malignant and aggressive BTCC was significant (*P *< 0.01), suggesting Apo-A1 could be a potential biomarker to classify BTCC in two-tie grading system. Using the cystoscopy as the gold standard for urinary sample categorization, ROC analyses rendered a diagnostic accuracy of 0.928 at an 18.22 ng/ml cutoff. Apo-A1 was also tested whether quantitative measurement could be utilized as a diagnostic tool to differentiate patients with low malignant BTCC and aggressive BTCC. Using the cystoscopy as the gold standard for urinary sample categorization, ROC analyses rendered a classified accuracy of 0.875 at a 29.86 ng/ml cutoff. These results indicated that Apo-A1 was detectable in the urine and supported the role of Apo-A1 to be a potential biomarker for diagnosis and classification of bladder cancer. To further validate above findings, 156 urine samples from healthy individuals or patients with urinary system diseases were randomly collected. Apo-A1 level was tested by ELISA. Clinical diagnosis and classification were made according to the cutoff values suggested by ROC analyses. Using the histopathological diagnosis after subsequent surgical interventions as the gold standard, Apo-A1 can distinguish between bladder cancer patients and controls with high sensitivity and specificity, which suggests the potential diagnostic application of urinary Apo-A1 for bladder cancer. Low malignant and aggressive BTCC were reported to develop and progress via two distinctive phenotypic pathways with drastically different biological behavior and clinical outcome, which determines that definite classification will be very helpful to decide medical treatment and predict the prognosis. The validation assay dealt with classification of bladder cancer further showed Apo-A1 can distinguish between low malignant and aggressive BTCC with high sensitivity and specificity. All of these findings strongly suggest the potential diagnostic utility of urinary Apo-A1.

Fibrinogen (fg), identified from three spots in our study, has been demonstrated to be associated with bladder malignancy and be a determinant in metastatic potential [26]. Fibrinogen Degradation Products (FDP) analysis in urine is rapid and the test is available clinically [22]. Several studies have shown that FDP is more sensitive than voided urine cytology for low grade tumors. However, the use of FDP as a tumor marker may be limited in that several studies have found FDP to be less specific than urine cytology, especially in the presence of benign genitourinary conditions [22,23]. LDHB, which was detected in BTCC urine samples of BTCC, is reported to be correlated with tumor expansion and invasion and patients with malignancies combined with increased serum LD isoenzymes activity usually have a poor prognosis [27]. LDHB could comparatively distinguish lung cancer from benign lung disease and healthy control and was correlated with the clinical stage of lung cancer [28], but there is no evidence to show LDHB has any connections with bladder cancer.

Recent study showed that urine CLU were significantly higher in individuals with bladder cancer with sensitivity of 49% and specificity of 92% respectively and suggested urine CLU could be a possible marker for bladder cancer. Our results are consistent with this. In our study, differentially expressed protein spot 12 was identified as CLU which was detected greatly increased in urine samples of patients with bladder cancer. Moreover, CLU was detected more strongly expressed in invasive BTCC compared with the expression in superficial BTCC and the pathologic stage and tumor grade showed close associations with CLU expression [29,30]. All these lines of evidence suggest that CLU might be considered as a potential diagnostic and prognostic biomarker for bladder cancer using urine, serum and/or molecular biology techniques. Over expression of Hp is very common in tumor cases. More and more evidence has showed that the levels of Hp in plasmas go up in many malignant tumors, such as pancreatic cancer, ovarian cancer and acute leukemia [31,32]. In our study, Spot 13 was identified as Hp α2 chain and it was found greatly increased in urine sample of patients with bladder cancer. Though maybe it does not belong to specific protein in bladder cancer, it could become an important check index for bladder cancer diagnosis and classification and improve the sensitivity and specificity of early diagnosis in lung cancer when combining with other diagnostic indexes.

## Conclusions

Taken together, our study indicates that the 2-DE proteomic analysis of urinary proteins is feasible for the diagnosis of bladder cancer as well as the classification in two-tie grading system. As a result of this approach, our study demonstrated that Apo-A1 protein levels in urine could serve as a biomarker for bladder cancer. Further, it can classify BTCC in two-tie grading system. Moreover, our results confirmed the importance of previously identified proteins and highlight new proteins that can add information regarding the pathophysiological mechanisms of bladder cancer.

## Methods

### Urinary Samples and Patients

Urine samples were collected with informed consents at the Department of Urology of the Second Hospital of Tianjin Medical University, including 24 volunteers with no evidence of disease and no history or evidence of urological cancer as normal controls, 8 volunteers with other chronic benign lesions in bladder, and 40 patients with BTCC. The average age of these three groups was 61.71 ± 8.35, 63.38 ± 4.93, and 63.00 ± 9.22, respectively. Patients were confirmed without resent PMH (post medical history) and any symptom of kidney failure. The presence of bladder cancer was confirmed by cystoscopy, together with histopathological information after subsequent surgical interventions. Tumors were graded according to the WHO criteria [33] and classified according to classification of two-tie grading system [14,15]. The number of patients with low malignant BTCC and aggressive BTCC was 24 and 16 respectively. Detailed clinical information was provided in Table 3. Urine samples were collected before the surgical operations and immediately centrifuged at 1500 × g for 5 min at 4°C to remove cell debris and particulate matter. 1 ml of supernatant was stored at -80°C for ELISA; the rest was employed for 2-DE sample preparation processing immediately. The detailed clinical information of patients providing urinary samples for Apo-A1 validation assay is not shown.

**Table 3:** Clinical information of patients providing urinary samples included for 2-DE analysis

No.	Age	Sex	Grade	Two-tie grading system	No.	Age	Sex	Condition
1	57	F	G2	Ⅰ	41	56	M	chronic inflammation of bladder
2	55	M	G2	Ⅰ	42	57	F	chronic inflammation of bladder
3	65	M	G1	Ⅰ	43	66	M	chronic inflammation of bladder
4	64	M	G2	Ⅰ	44	65	F	bladder atypia hyperplasy
5	69	F	G2	Ⅰ	45	70	M	bladder atypia hyperplasy
6	66	M	G2	Ⅰ	46	61	M	bladder atypia hyperplasy
7	67	F	G2	Ⅰ	47	67	M	bladder atypia hyperplasy
8	75	F	G2	Ⅰ	48	65	M	inverted papilloma of bladder
9	51	M	G1	Ⅰ	49	50	F	normal
10	69	F	G1	Ⅰ	50	53	F	normal
11	60	M	G1	Ⅰ	51	56	F	normal
12	62	M	G1	Ⅰ	52	58	F	normal
13	60	M	G2	Ⅰ	53	70	M	normal
14	61	M	G2	Ⅰ	54	72	M	normal
15	69	M	G2	Ⅰ	55	61	M	normal
16	67	F	G1- G2	Ⅰ	56	67	M	normal
17	61	M	G2	Ⅰ	57	59	M	normal
18	79	M	G2	Ⅰ	58	73	M	normal
19	61	M	G1- G2	Ⅰ	59	75	M	normal
20	59	F	G2	Ⅰ	60	64	M	normal
21	54	M	G2	Ⅰ	61	58	M	normal
22	48	M	G2	Ⅰ	62	52	M	normal
23	56	M	G1- G2	Ⅰ	63	72	M	normal
24	57	F	G2	Ⅰ	64	51	M	normal
25	71	F	G2-G3	Ⅱ	65	50	M	normal
26	80	F	G2-G3	Ⅱ	66	57	F	normal
27	76	M	G3	Ⅱ	67	63	F	normal
28	53	M	G3	Ⅱ	68	65	F	normal
29	52	M	G3	Ⅱ	69	54	F	normal
30	59	M	G3	Ⅱ	70	58	F	normal
31	69	M	G3	Ⅱ	71	77	F	normal
32	60	M	G3	Ⅱ	72	66	M	normal
33	58	M	G3	Ⅱ				
34	39	M	G3	Ⅱ				
35	80	M	G3	Ⅱ				
36	55	M	G3	Ⅱ				
37	68	M	G3	Ⅱ				
38	72	M	G3	Ⅱ				
39	77	M	G3	Ⅱ				
40	59	M	G3	Ⅱ				

Ⅰ: Low Malignant BTCC; Ⅱ: Aggressive BTCC

### Sample preparation for 2-DE

Urine supernatant mixed with ethanol at a ratio of 3:1. The mixture was incubated at 4°C for 10 min. The precipitant was collected by centrifuging at 12,000 × g for 5 min at 4°C and washed twice using 25% ethanol. The pellet was resuspended in solubilizing buffer which contained 7 M urea, 2 M thiourea, 4% CHAPS, 0.2% ampholytes, 65 mM DTT. The concentration of urinary proteins was determined by using the RC DC protein assay kits (Bio-Rad). Inorganic salts and any other interfering components were removed by using 2D Clean-Up Kit (Bio-Rad).

### 2-DE and stain

Urine samples were diluted in 350 μl rehydration buffer containing 7 M urea, 2 M thiourea, and 4% CHAPS, 0.2% (v/v) ampholytes (pH 3-10), 65 mM DTT, 2 mM tributylephosphine (TBP) and a trace of bromophenol blue. Diluted samples were loaded onto IPG strips, 17 cm pH 4-7 L, for the first dimension. Strips were covered with mineral oil and passively rehydrated overnight. IEF was performed at voltage linearly increased from 150 to 8000 V during the first 10 h, followed by 10,000 V for a total of 75,000 Vh. Before the second-dimensional run, IPG strips were equilibrated with the buffer containing 6 M urea, 2% SDS, 0.375 M Tris-HCl pH 8.8, 20% glycerol, 2% DTT for 15 min and equilibrated further with the same buffer additionally containing iodoacetamide (2.5%) instead of DTT for 15 min within the strip tray. The IPG strips were then placed onto a 12% polyacrylamide gel and covered with 0.5% agarose gel. The second dimension was run at 30 mA for 4.5 h. Polyacrylamide gels were stained with CBB G-250 and/or silver according to the procedures described previously [34]. Each sample was used in triplicates. The resulting 2-DE images were analyzed using the PDQuest software program.

### Identification of Proteins by MALDI-TOF/TOF-MS

Significantly differed protein spots were excised and destained with a solution of 25 mM ammonium bicarbonate and 50% ACN. The gels were then dried completely by centrifugal lyophilization. An in-gel digestion was performed with 0.01 μg/μl trypsin (Promega) in 25 mM ammonium bicarbonate for 15 h at 37°C. The supernatants were collected, and the tryptic peptides were extracted from the gel sequentially with 5% TFA at 40°C for 1 h and with a solution of 2.5% TFA and 50% ACN at 30°C for 1 h. The extracts were pooled and dried completely by centrifugal lyophilization.The peptide mixtures were redissolved in 0.5% TFA, and 1 μl of the peptide solution was mixed with 1 μl of matrix (4-hydroxy-a-cyanocinnamic acid in 30% CAN and 0.1% TFA) before spotting on the target plate. MALDI-TOF/TOF-MS analysis of the samples was carried on a mass spectrometer 4700 (Applied Biosystems, Framingham, MA) in a positive ion reflector mode. Following MALDI-TOF/TOF, the instrument was switched to MS/MS mode, and the five strongest peptides from the MS scan were isolated and fragmented by collision-induced dissociation with air. The ion acceleration voltage was 20 kV. The obtained peptide mass fingerprinting data were further processed using the GPS Explorer software (Applied Biosystems) that acts as an interphase between the Oracle database containing the raw spectra and a local copy of the MASCOT search engine. The Homo sapiens subsets of the sequences in the Swiss-Prot and NCBI nonredundant protein sequence databases were utilized for MASCOT searches. The following search parameters were used in all MASCOT searches: limited protein molecular weight from 700-4000 Da; peptide mass tolerance of ± 0.1 Da; tolerance of one missed trypsin cleavages; a maximum error tolerance of ± 2.0 Da in the MS data and ± 0.6 Da in the MS/MS data; protein hits with more than five significant matched peptides with the distinct sequences. A mass accuracy between 35 and 100 ppm (Swiss-Prot Database) and between 42 and 100 (NCBI Database) were statistically considered to estimate the confidence of protein identifications.

### Western blot

Total urinary protein of healthy volunteers and patients with bladder cancer was determined by RC DC protein assay kits using BSA as the standard (Bio-Rad) and equal protein amount (25 μg) was loaded onto 10% SDS-PAGE gels. Subsequently, the electrophoresed proteins were transferred to a PVDF membrane (Bio-Rad). After blocking, the membrane was incubated with primary antibodies (Fb, F4639; LDHB, WH0003945M1; Apo-A1, WH0000335M1; CLU, WH0001191M1; Hp, H6395; all from Sigma) for 2 h at room temperature. Following washing, the membrane was exposed to the secondary antibodies conjugated with horseradish peroxidase for 30 min. The antigen-antibody complex was detected by enhanced chemiluminescence using ECL reagents (Thermo Fisher Scientific) and visualized by autoradiography. The X-ray films were digitalized for subsequent densitometric quantification of the bands using the morphometric program Optimas ( Optimas Corporation, Seattle, USA).

### Enzyme linked immunosorbent assay (ELISA)

Soluble urinary Apo-A1 concentrations were measured by ELISA using Quantikine Immunoassay Kits (ADL). Urine samples were centrifuged for 5 min at 1500 rpm and the supernatants were utilized for ELISA. ELISA was performed according to the methods recommended by the manufacturer. After the development of the colorimetric reaction, the OD at 450 nm was quantified by an eight-channel spectrophotometer, and the OD readings were converted to nanograms per milliliter (ng/ml) on the basis of the standard curves obtained with Apo-A1 standard preparation in assay. Each sample was tested in duplicate. Apo-A1 concentrations were represented as mean ± SD.

### Statistical Analysis

Urine samples (n = 72) were utilized to analyze the clinical utility of Apo-A1 at discriminating patients with bladder cancer from healthy individuals and discriminating patients with low malignant BTCC from aggressive BTCC. Urine samples categorization was based on cytology and cystoscopic observations, the latter being utilized as the "gold standard". Receiving operating curve (ROC) analyses were used to define the most optimal diagnostic cutoff as well as the diagnostic performance given by AUC, estimating its 95% confidence interval at optimal cutoffs. Statistical analyses were performed using the SPSS statistical package (version 13.0).

## Abbreviations

**BTCC**: bladder transitional cell carcinoma; **Fb**: fibrinogen; **LDHB**: lactate dehydrogenase B; **Apo-A1**: apolipoprotein-A1; **CLU**: clusterin; **Hp**: haptoglobin; **2-DE**: two-dimensional electrophoresis; **MS**: mass spectrometry; **CBB**: coomassie brilliant blue; **AUC**: area under the curve; **ROC**: receiver operating curve.

## Competing interests

The authors declare that they have no competing interests.

## Authors' contributions

HL and CL conceived the idea of proteomics study, participated in its design and performed the most of the experiments. HW and SW helped on 2-DE performance. TZ and JW carried out sample collection. JC conceived and supervised the study. All authors read and approved the final manuscript.
